# Hypertension: A Behavioral Medicine Perspective

**DOI:** 10.1155/2012/385690

**Published:** 2012-04-22

**Authors:** Tavis Campbell, Simon L. Bacon, Joel E. Dimsdale, Douglas Carroll

**Affiliations:** ^1^Department of Psychology, University of Calgary, Calgary, AB, Canada T2N 1N4; ^2^Department of Exercise Science, Concordia University, Montreal, QC, Canada H4B 1R6; ^3^Research Centre, Hopital du Sacre-Coeur de Montreal, Montreal, QC, Canada H4J 1C5; ^4^Research Centre, Montreal Heart Institute, Montreal, QC, Canada H1T 1C8; ^5^University of California, San Diego, La Jolla, CA 92037-0603, USA; ^6^Applied Psychology, University of Birmingham, Birmingham B15 2TT, UK

Behavioral medicine is an interdisciplinary field concerned with the development and integration of behavioral and biomedical science, knowledge, and techniques relevant to health and illness and the application of this knowledge and these techniques to prevention, diagnosis, treatment, and rehabilitation. From a behavioral medicine standpoint, hypertension is a lifestyle disorder, aggravated by unhealthy diet, adiposity, excessive alcohol intake, and stress. The World Health Organization has identified hypertension as the number one risk factor for mortality worldwide [1]. Although considerable progress has been made in developing pharmacological treatments for hypertension, the underlying behaviors that contribute to the majority of hypertension in the first place (and which are also related to most chronic illnesses) continue to escalate. For example, in the USA, the Centers for Disease Control have been tracking increases in overweight and obesity on an annual level, and current estimates suggest that 33.8% [2, 3] of the US adult population is obese. Perhaps more troubling are the statistics for children in the USA, of whom 17% obese—nearly 3 times the number in 1980 [4]. Although there is clear consensus on the impact of behaviors such as exercise and nutrition on blood pressure, relatively little is known about how to motivate people to take up and consistently engage in these health behaviors. This is a striking knowledge gap because the first line of therapy for the treatment of hypertension is usually a recommendation that the patient modifies his/her lifestyle by, for example, changing diet or increasing physical activity. Despite considerable investigation, even less is known about how stress affects physiology to contribute to the hypertensive process or how stress affects blood pressure through effects on health behaviors. While there is substantial work left to be completed in behavioral medicine, we should, at the same time, recognize the progress the field has made in elucidating mechanisms through which lifestyle contributes to hypertension and in developing behavioral interventions for disease management.

The main focus of this special issue is to highlight the role of behavioral and psychological factors in the etiology, prevention, and treatment of hypertension. Two papers review mechanisms through which biobehavioral factors influence and are affected by increases in blood pressure. J. R. Jennings and A. F. Heim argue that findings from human neuroimaging suggest the brain is an early target for hypertension. W. Gerin and colleagues propose a model to explain the effects of cumulative exposure to stress (including ruminative stress) that leads, over time, to an upward resetting of the resting blood pressure, resulting in an increased risk of hypertension.

Another five papers in the issue examine the impact of sleep, anxiety, anger, depressed mood, and various health behaviors on blood pressure, blood pressure regulation, and endothelial function. These papers provide an excellent sense of current investigations into the impact of various candidate psychological/behavioral factors on hypertension, as well as some of the intricacies and challenges involved in deriving clinically meaningful conclusions from this work.

The issue shifts focus to consider the impact of psychological interventions on blood pressure, with a review of a stress management intervention, meditation. While meditation techniques appear to produce small yet meaningful reductions in blood pressure, the review also points to serious methodological shortcoming and the need to attain a higher standard of quality, with more randomized controlled trial studies. Another paper suggests stress-activated gene × environment interactions may contribute to individual variability in blood pressure reductions from behavioral interventions.

A series of papers consider the uptake of behavioral medicine interventions, including self-monitoring of blood pressure in hypertension, a relatively simple and easily adopted activity that is associated with small but meaningful clinical improvements. The special issue ends with a provocative review of behavioral versus public health efforts to curb an obvious target for health behavior change, sodium consumption.

In highlighting some of the key areas of endeavor in the field of behavioral medicine, we hope to encourage readers to incorporate psychological and behavioral variables into their research on hypertension. Given the web of underlying causality, we believe that such an approach has considerable potential to inform efforts aimed at better understanding the causes, consequences, and treatment of hypertension.

*Tavis Campbell* *Tavis Campbell* *Simon L. Bacon* *Simon L. Bacon* *Joel E. Dimsdale* *Joel E. Dimsdale* *Douglas Carroll* *Douglas Carroll*
